# *CXCR5* Regulates Neuronal Polarity Development and Migration in the Embryonic Stage *via* F-Actin Homeostasis and Results in Epilepsy-Related Behavior

**DOI:** 10.1007/s12264-023-01087-w

**Published:** 2023-07-17

**Authors:** Zhijuan Zhang, Hui Zhang, Ana Antonic-Baker, Patrick Kwan, Yin Yan, Yuanlin Ma

**Affiliations:** 1 Department of Neurology, Chongqing Key Laboratory of Neurology, The First Affiliated Hospital of Chongqing Medical University, Chongqing, 400016 China; 2 Chongqing Emergency Medical Center, The First Affiliated Hospital of Chongqing Medical University, Chongqing, 400016 China; 3 Department of Neuroscience, Central Clinical School, Monash University, Melbourne, Australia

**Keywords:** Epilepsy, *CXCR5*, Embryonic neurogenesis, Pluripotent stem cells, Intrauterine electroporation, F-actin, Neuronal polarity, Neuronal migration

## Abstract

Epilepsy is a common, chronic neurological disorder that has been associated with impaired neurodevelopment and immunity. The chemokine receptor *CXCR5* is involved in seizures *via* an unknown mechanism. Here, we first determined the expression pattern and distribution of the *CXCR5* gene in the mouse brain during different stages of development and the brain tissue of patients with epilepsy. Subsequently, we found that the knockdown of *CXCR5* increased the susceptibility of mice to pentylenetetrazol- and kainic acid-induced seizures, whereas *CXCR5* overexpression had the opposite effect. *CXCR5* knockdown in mouse embryos *via* viral vector electrotransfer negatively influenced the motility and multipolar-to-bipolar transition of migratory neurons. Using a human-derived induced an *in vitro* multipotential stem cell neurodevelopmental model, we determined that *CXCR5* regulates neuronal migration and polarization by stabilizing the actin cytoskeleton during various stages of neurodevelopment. Electrophysiological experiments demonstrated that the knockdown of *CXCR5* induced neuronal hyperexcitability, resulting in an increased number of seizures. Finally, our results suggested that *CXCR5* deficiency triggers seizure-related electrical activity through a previously unknown mechanism, namely, the disruption of neuronal polarity.

**Supplementary Information:**

The online version contains supplementary material available at 10.1007/s12264-023-01087-w.

## Introduction

Epilepsy is a common and severe chronic neurological disorder [1]. Approximately 90% of patients with epileptic encephalopathy have uncontrolled seizures despite treatment with multiple antiepileptic drugs, and ~50% exhibit cortical dysplasia [2]. Focal cortical dysplasia is caused by impaired neuronal migration or cell proliferation in the cerebral cortex and is one of the most frequent causes of refractory epilepsy [3]. Cortical dysplasia is present in up to 75% of cases of surgically-treated childhood epilepsy and ~20% of cases of adult epilepsy [4]. Brain development, which begins during embryogenesis, is tightly regulated by precisely-timed gene expression patterns. An increasing number of genetic abnormalities have been reported to negatively affect neurogenesis and higher brain functions in adulthood, resulting in a range of neuropsychiatric disorders such as epilepsy, autism, and schizophrenia [5, 6]. The mechanisms underlying the pathology of epilepsy have been widely explored, with research increasingly pointing to chemokine receptors as causative factors. For instance, genetic analysis in mice has demonstrated that reductions in chemokine receptor levels alter the migration of interneurons and modulate key molecular signaling pathways, thereby disrupting the development of cortical circuits [7]. Embryonic and adult neural progenitors exhibit broad sensitivity to chemokines, which supports the hypothesis that chemokines play an important role in controlling progenitor cell migration in both the embryonic and adult brain [8]. Chemokine receptors and neurogenesis are interdependent [9]. The classical chemokine receptor *CXCR5* functions in cell migration and localization and coordinates intercellular interactions [10–12]. *CXCR5* expression is elevated in the brain tissue of patients with epilepsy [13]. The role of *CXCR5* in brain development has been increasingly studied over recent years, with studies showing that *CXCR5* is involved in the regulation of neural stem cell (NSC) and neuronal regeneration in the mouse brain [14, 15], neuronal differentiation [16, 17], neurogenesis, gliogenesis, and synaptogenesis [16, 18]. These findings suggest that *CXCR5* dysregulation during neural development may underlie the pathology of epilepsy, an idea that merits further investigation [19].

In this study, we focused on the effects of *CXCR5* in the development of epilepsy. First, we clarified the expression and subcellular localization of CXCR5 at different developmental stages of the murine brain and induced pluripotent stem cell (iPSC) culture. Protein profiling suggested that CXCR5 interacts with numerous cytoskeletal proteins and is closely associated with actin. Next, we elucidated the effects of knocking down or overexpressing *CXCR5* on seizures in two animal models of epilepsy. Furthermore, we applied intrauterine electroporation to determine the effect of *CXCR5* deficiency and overexpression on neuronal migration in late gestational embryonic mice and tracked the morphological changes occurring in the development of individual neurons *in vitro* through a human-derived iPSC neurodevelopmental model. Finally, we used electrophysiological techniques to probe the effects of altered *CXCR5* activity on the intrinsic excitability of neurons. In summary, our results provide a clearer understanding of the expression of *CXCR5* by brain region and provide a foundation for exploring its role in epilepsy as well as the putative underlying mechanisms.

## Materials and Methods

### Animals

The experimental mice (C57BL/6J) were provided by the Animal Experimentation Center of Chongqing Medical University and were cared for under the following conditions: temperature (22°C–24°C); relative humidity (50% ± 1%); 12 h light cycle beginning at 08:00; standard access to food and water. All experimental animal care conditions were strictly in accordance with the Chongqing Medical University Institutional Animal Care and Use Guidelines and approved by the Animal Care Committee. The mice were used for mating and breeding at 6–8 weeks, 1 male and 2 females per cage. Cages were closed at 20:00 the previous day, and vaginal plugs were tested at 08:00 the next day; mice with positive plugs were defined as embryonic day 0.5 (E0.5), mice were defined as E19 or P0 days on the day of birth, and adults were P60 days. All animal experiments complied with the ARRIVE guidelines and were carried out in accordance with the U.K. Animals (Scientific Procedures) Act, 1986, and associated guidelines, EU Directive 2010/63/EU for animal experiments.

### Cells

The First Affiliated Hospital of Chongqing Medical University provided us with ethically-approved human-derived skin fibroblasts for experimental studies. Experiments were conducted in accordance with the principles of the Declaration of Helsinki and approved by the Ethics Committee of the First Affiliated Hospital of Chongqing Medical University.

See Table S1 for specific information on plasmid vectors, viral vectors, antibodies, and reagents.

### Western Blot Experiment

We selectively mated 6- to 8-week-old female and male mice (C57BL/6J), isolated [20] cortical tissue from embryonic and postnatal mice at different developmental time points (E14.5, E17.5, P0, P7, P14, P21, and P60), and extracted total protein samples for Western blotting experiments. Protein samples were separated by 12.5% SDS-polyacrylamide gel electrophoresis, and the gels were separated and electrically transferred to 0.22-μm PVDF membranes. The membranes were blocked with 10% skim milk powder containing tris-buffered saline with 0.1% Tween 20 (TBST) for 1 h at room temperature and then incubated with the noted CXCR5 and CXCL13 antibody/β-tubulin antibody overnight at 4°C. The membranes were washed 3 times with TBST for 10 min each and then incubated in anti-mouse or anti-rabbit secondary antibody containing horseradish peroxidase at room temperature for 1 h. The membranes were then washed three times with TBST and tagged using enhanced chemiluminescence reagents (Thermo Fisher, MA), and a Fusion FX5 image analysis system (Vilber Lourmat, Marne-la-Vallée, France) was used to visualize the bands. Relative protein levels were determined by normalizing the signals for CXCR5 to β-tubulin levels and CXCL13 to β-tubulin and CXCR5, p-Cofilin, Cofilin to actin, and p-Cofilin / Cofilin and F-actin/G-actin using Quantity One software (Bio-Rad, CA).

### F-actin and G-actin Protein Extraction

Tissue proteins in five groups (Con-shRNA, AAV-*shCXCR5*, Con-AAV-*CXCR5*, AAV-*CXCR5*, and AAV-*shCXCR5* + Jasplakinolide) were extracted at E19 to measure the ratio of F-actin to G-actin contents (F-actin/G-actin), which were determined by Western blot. Since F-actin is insoluble and G-actin is soluble, these tissue protein lysates were centrifuged at 15000×*g* for 30 min, the soluble protein was extracted from the supernatant (labeled G-actin protein), and the precipitated protein was F-actin. Then we re-suspended the precipitated protein in an equivalent volume of lysis buffer and incubated it on ice for an hour, gently mixing every 5 min to promote protein dissolution, centrifuged this buffer at 15,000 for 30 min again, and labeled F-actin protein. The western blot method was applied as above.

### Immunoprecipitation

Cortical tissue from mice at different developmental stages (E14.5, E17.5, P0, P7, P14, P21, and P60) was used for immunoprecipitation. Tissue lysates were first incubated with 30 µL protein A/G magnetic beads for 2 h at 4°C to reduce non-specific binding. Then 0.5 µg rabbit IgG, 2 µg CXCR5 antibody, 2 µg actin antibody, or 2 µg magnetic beads were incubated with 40 µL protein A/G for 2 h at 4°C and washed four times with PBS (0.05% PBST), using 0.05% Triton X-100. The pretreated protein samples were then mixed with antibody-protein A/G-magnetic bead complexes, incubated at 4°C for 2 h, and washed four times with 0.05% PBST. Protein samples were mixed with 1× loading buffer and heated to 95°C for 10 min for denaturation and elution. After magnetic separation, protein samples were collected for Western blot analysis.

### Epilepsy-Induced Models

#### Pentylenetetrazol (PTZ)-Induced Acute Model

Previously, we had administered four viruses to embryonic mice (E14.5), and selected male mice with effective injections were carefully raised to adulthood (P60), and a subthreshold dose of pentylenetetrazol (35 mg/kg) was injected intraperitoneally into each group (4 groups of 10 mice each) at regular intervals of 24 h every day for 15 days (all injection times were between 09:00 and 12:00 Beijing time). Mice were observed for at least 30 min after each PTZ injection, and seizures were recorded using the Racine criteria [21]. with the following scores: Grade 0, no response; Grade 1, gaze and reduced movement; Grade 2, activation of extensor muscles and rigidity; Grade 3, repetitive head and limb movements; Grade 4, continuous forelimbs with clonus; and Grade 5, generalized tonic-clonic seizures (GTCSs) with postural reflex loss and death. As a result of the susceptibility to death during a GTCS, we also calculated the survival rate of the mice in each group.

#### KA-Induced Animal Model

Simultaneously, we also administered four viruses to embryonic mice (E14.5), carefully raised them to adulthood, selected male mice (P60) with effective injections, and created our second epilepsy animal model. KA was injected stereotaxically into the CA1 region of the hippocampus of each group (4 groups, 15 mice per group; only one KA injection per mouse), and the mice were closely observed for 2 h after the injection. Once a sustained status epilepticus (SE) seizure was detected, the sedative diazepam was immediately administered intraperitoneally, and only mice that were confirmed to have SE seizures and recovered from the intraperitoneal diazepam injection were returned to the cage for follow-up video monitoring (we ensured that no less than 10 mice in each group survived for follow-up statistical analysis and local field potential (LFP) recording).

We performed continuous video surveillance for 1–4 weeks after successful KA injection as an induced epilepsy model to assess the formation of spontaneous recurrent seizures (SRSs) with statistical analysis.

#### LFP Recording

Two stainless steel screws were implanted as grounding screws in the anterior skull, and platinum-iridium alloy microfilaments (25 μm diameter; Plexon, Dallas, TX) were implanted on both sides of the dorsal hippocampus (from bregma (in mm): AP: −1.6; ML: ±1.6; DV: −1.5). The guide cannula, microfilaments, and U-shaped frame for head fixation were secured to the skull; 1 month later we made KA localization injections to successfully induce SE, LFPs were recorded for 2 h [22]. In summary, the heads of conscious mice were immobilized to minimize behavioral state-induced changes in LFP. LFP was recorded using a MAP data acquisition system (Plexon, Dallas, TX) with signals filtered (0.1 to 500 Hz), pre-amplified (1000×), and digitized at 4 kHz; LFP data were examined using NeuroExplorer (Nex Technologies, Littleton, MA). A set of spontaneous paroxysmal discharges with high amplitude spike activity of >2 SD from baseline and frequencies >1 Hz were defined as SLEs if they lasted 5 s or longer. SLEs were also confirmed by spectrogram analysis.

### Intrauterine Electroporation and Virus Injection in the Cerebral Cortex of Mouse Embryos

#### Plasmid Transfection

Pregnant mice at E14.5 were anesthetized with 0.7% pentobarbital sodium. The abdomen was then sterilized, the uterus was exposed by incision, and a high concentration (2 µg/µL) of the *CXCR5* knockdown plasmid, the knockdown control plasmid, the *CXCR5* overexpression plasmid, or the overexpression control plasmid was injected into the lateral ventricles of intrauterine embryos through polished glass micropipettes. Then, CUY650P5 electrodes were placed on both sides of the fetal mouse head, and the positive electrode was placed tangentially in the target cortical area, followed by electroporation (CUY21, Nepa Gene, Ichikawa-City, Chiba, Japan) (pulse voltage: 33 V, pulse duration: 50 ms, pulse interval: 950 ms, number of pulses: 4). Finally, the uterus was returned to the abdominal cavity and the abdomen was sutured. After birth (E19/P0), brain slices were isolated and processed.

#### Virus Infection

Pregnant mice at E14.5 were anesthetized with 0.7% pentobarbital sodium, the abdomen was sterilized, an incision was made to expose the uterus, and viral vectors (Con-shRNA, AAV-*shCXCR5*, Con-AAV-*CXCR5*, and AAV-*CXCR5*) were injected into the lateral ventricles of intrauterine embryos. Then, the uterus was returned to the abdominal cavity, and the abdominal wall and skin were sutured. The dark green crescent shape characteristic of the lateral ventricle observed after injection indicated that the injection was successful. The mice were carefully raised to adulthood (P60) for the generation of animal models of epilepsy and behavioral experiments.

### Immunostaining of Brain Slices

Mice that had undergone intrauterine electroporation were anesthetized by intraperitoneal injection of 1% pentobarbital (E19) and embryonic brain tissue was collected and fixed in 4% paraformaldehyde (PFA) for 24 h, followed by cryopreservation in a graded series of sucrose concentrations (15%, 20%, and 25%). After embedding in the optimal cutting temperature (OCT) compound, brain tissue was snap-frozen in isopentane at −70°C. Coronal sections were cut at 20 µm–25 µm on a Leica CM1950 cryostat, washed twice (10 min each) with PBS to remove the OCT, and permeabilized with 3% BSA and 0.3% Triton X-100 in PBS for 30 min at room temperature. The sections were then directly transferred into a solution containing the primary antibody (anti-GFP or anti-mCherry), incubated overnight at 4°C, washed twice (10 min each) with PBS, and incubated with the designated concentrations of the secondary antibody and Hoechst for 1 h at room temperature. Finally, the sections were washed twice with PBS (10 min each), covered with antifade mounting medium, and coverslipped. Images were captured under a laser-scanning confocal microscope. Brain sections obtained at different developmental stages (E17.5, P7, and P60) were stained with fluorescently-labeled primary antibodies (anti-MAP2, anti-GFAP, and Map2/CXCR5 or GFAP/CXCR5 co-staining) using the same method.

### Cell Culture

We used healthy, adult-derived fibroblasts cultured and maintained in an F12 medium. IPSCs were generated from the fibroblasts using the Stemgent StemRNA 3rd Gen Reprogramming Kit for Reprogramming Adult and Neonatal Human Fibroblasts and Lipofectamine RNAiMax Transfection Reagent following the manufacturer’s instructions. The fibroblasts were grown in matrix-coated six-well plates at 37°C with 5% CO_2_ until the growth of pluripotent stem cell clones was observed and good-quality clones were hand-picked for all subsequent experiments. Pluripotency was verified by immunofluorescence staining using anti-OCT4 and anti-SSEA4 antibodies.

IPSCs at ≥80% confluence were selected for the induction of ectodermal differentiation. After 7 days of continuous induction (the medium was refreshed every 2 days), the medium was changed to neural progenitor cell expansion medium, and the cells were cultured for another 7 days until a tendency for spontaneous differentiation was observed. The cells were transferred to low-adherence 96-well culture plates for suspension culture, imaged under a light microscope, and immunostained with fluorescently labeled anti-PAX6 and anti-Nestin antibodies for ectodermal verification.

### Neural Stem Cells

Quality NSCs are round-shaped spheres that proliferate rapidly. These spheres were dissociated with Accutase Cell Detachment Solution and inoculated onto Laminin/PDL double-coated culture plates and switched to NPM medium for maintenance with the addition of virus (Con-shRNA, AAV-*shCXCR5*, Con-AAV-*CXCR5*, and AAV-*CXCR5*). The day of the initial addition of the virus was considered day 0 (DIV0). The development from neural progenitor cells to naïve neurons, and finally to mature neurons was observed and the details were recorded. In addition, BrainPhys Neuronal Maturation Medium must be added gradually and neurons can be further matured in culture for up to 60 days. Neurons were identified by immunofluorescence staining with anti-MAP2, anti-βIII-tubulin, anti-MAP2/anti-CXCR5 (co-staining), and anti-Na^+^/K^+^-ATPase/anti-CXCR5 (co-staining) antibodies to determine sub-localization in the cells. DIV4 and 8-week-old neurons were selected for immunofluorescence staining (anti-GFP and anti-mCherry antibodies) for morphological observation and statistical analysis.

### Immunofluorescence and Morphological Analysis

To assess neuronal growth at different stages and determine the localization of CXCR5 *in vitro*, the cells were subjected to immunofluorescence staining at different stages of differentiation. DIV4 and 8-week-old neurons were fixed in 4% PFA for 30 min at 37°C, washed three times in PBS (5 min each), permeabilized with 0.1% Triton X-100 for 30 min, blocked with goat serum for 1 h, and incubated with a combination of primary antibodies targeting GFP, mCherry, Nestin, PAX6, and Na^+^/K^+^-ATPase overnight at 4°C. After washing in PBS, the cells were incubated with the corresponding secondary antibodies for 1 h at room temperature in the dark. Images were captured by fluorescence microscopy. The number of primary neuronal protrusions (protrusions directly connected to the neuronal cytosol), secondary neuronal protrusions, and axon length were determined. Sholl analysis was used to assess neuronal dendritic branching patterns and the number of intersections between neurons and the neuronal cytosol was counted and plotted. The cytoskeletal analysis involved counting the total number and the total length of dendritic branches.

### Whole-Cell Current-Clamp Recordings

Whole-cell current-clamp recordings were performed. Borosilicate glass electrodes (resistance 5–15 mΩ) were filled with a solution of (in mmol/L)135 potassium gluconate, 7 NaCl, 10 HEPES, 2 Na_2_ATP, 0.3 Na_2_GTP, and 2 MgCl_2_ (pH 7.4). Recordings were made at room temperature using a MultiClamp 700B amplifier (Molecular Devices, San Jose, CA, USA). Cells were continuously perfused with oxygenated artificial cerebrospinal fluid (in mmol/L; 125 NaCl, 25 NaHCO_3_, 1.25 NaH_2_PO_4_, 3 KCl, 2 CaCl_2_, 1 MgCl_2_, 25 glucose, and 3 pyruvate bubbled with 95% O_2_/5% CO_2_.

To record evoked action potentials (eAPs), glass pipette electrodes were filled with an internal solution of the following composition (in mmol/L): 60 K_2_SO_4_, 40 HEPES, 4 MgCl_2_·6H_2_O, 60 N-methyl-d-glutamine, 0.5 BAPTA, 12 phosphocreatine, 2 Na_2_ATP, and 0.2 Na_3_GTP. Neuronal eAPs at resting membrane potential were recorded in whole-cell patch-clamp current-clamp mode.

### Phalloidin Labeling

Neurons were stained with phalloidin in combination with anti-GFP and anti-mCherry primary antibodies as described above. Anti-phalloidin secondary antibodies were adjusted according to the color of the fluorescent protein. For example, for the knockout group (AAV-EGFP labeled in fluorescent green), the phalloidin-conjugated secondary antibody was red, and *vice versa*.

### Statistical Analysis

Experimental data are expressed as the mean ± SEM, and Student’s *t*-test for independent samples or Mann-Whitney U was used for comparisons between two groups. One-way ANOVA or repeated-measures ANOVA or the Kruskal–Wallis test was used for comparisons between multiple groups. The Kaplan-Meier method and log-rank test were used for survival analysis, and *P* <0.05 was considered a statistically significant difference. ImageJ software was used for morphological statistics and fluorescence quantification of neurons, and GraphPad Prism 9.0 was used for graphing. Adobe Photoshop software was used for image processing.

### Protein Spectroscopy Analysis

Proteomic analysis of isolated brain tissue (E17.5) was applied using non-targeted liquid chromatography-mass spectrometry (Luming Biological Technology, Shanghai, China, http://www.lumingbio.com/). The five factors we used to evaluate potential interacting proteins were the number of peptides, peptide spectrum matches (PSMs), unique peptides, coverage, and score of peptides matches to proteins, obtained 215 potential proteins.

## Results

### Localization and Expression of CXCR5 in the Mouse Brain (C57BL/6J) at Different Developmental Stages and in Brain Tissue from Patients with Epilepsy

**Fig. 1:**
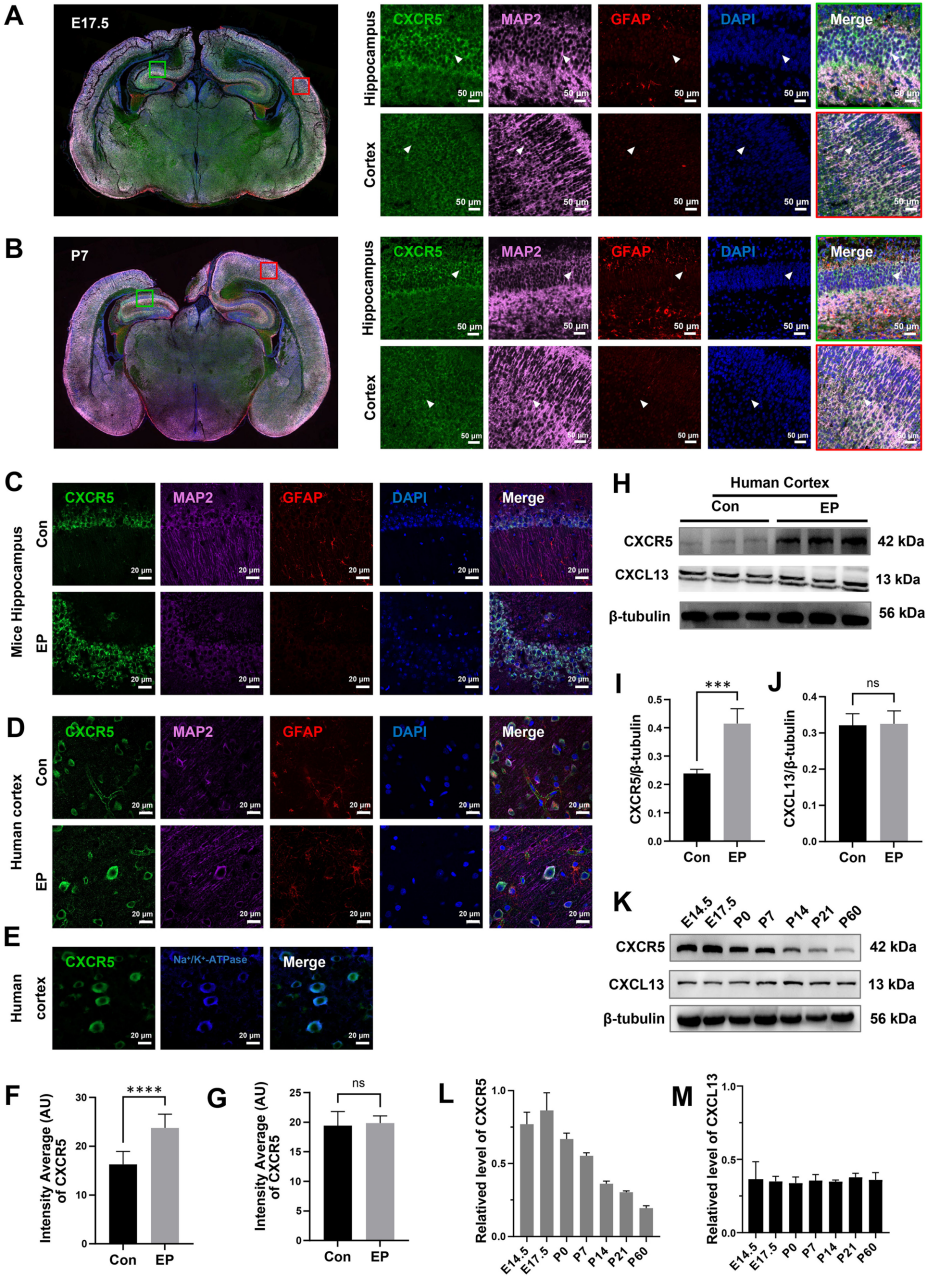
Localization and expression of CXCR5 in a C57BL/6J mouse and epileptic tissue. **A** Distribution of CXCR5 in C57BL/6J mouse at E17.5; the red box is in the cortex, and the green box is in the hippocampus. Co-localization of CXCR5 with MAP2 but not with GFAP. Scale bars, 50 μm. **B** Distribution of CXCR5 in a C57BL/6J mouse at P7; the red box is in the cortex, and the green box is in the hippocampus. Co-localization of CXCR5 with MAP2 but not with GFAP. Scale bars, 50 μm. **C** Distribution of CXCR5 in mouse hippocampus. Co-localization of CXCR5 with MAP2 but not with GFAP. Scale bars, 20 μm. **D** Localization of CXCR5 in the human-derived cortex, consistent with the results in mice. Scale bars, 20 μm. **E** CXCR5 is co-localized with Na^+^/K^+^-ATPase, and is mainly expressed in the neuronal membrane. **F** Statistics of CXCR5 fluorescence in epilepsy patients and the control group. **G** Statistics of CXCR5 fluorescence in epileptic mouse hippocampus and the control group. **H** Western blot images of CXCR5 and CXCL13 in the human cortex (epilepsy patients) and the control group. **I** Protein levels of CXCR5/β-tubulin. **J** Protein levels of CXCL13/β-tubulin. **K** Western blot images of CXCR5 and CXCL13 at different developmental stages in mice (E14.5, E17.5, P0, P7, P14, P21, and P60). **L** Protein levels of CXCR5/β-tubulin, showing that expression peaks at E17.5 and declines postnatally. **M** Protein levels of CXCL13/β-tubulin, showing that they remain unchanged at different developmental stages in mice (E14.5, E17.5, P0, P7, P14, P21, and P60). The data are presented as the mean ± SEM. Student’s *t*-test was used for **I**, **J**, **F**, **G**. *n* = 3 in **F**, **G**, **I**, **J**, **L**, and **M**. ***P <0.005; ****P <0.0001; ns, no significant difference.

We first observed the distribution of CXCR5 in C57BL/6J mice at different stages of development. At E19 and P7, CXCR5 was found to co-label with MAP2, but not GFAP (Fig. 1A, B), in the murine hippocampus and cortex; the same results were obtained at adulthood (P60) (Fig. 1C). Secondly, we found that CXCR5 expression was increased in the cortex of humans with epilepsy but unchanged in the hippocampus of mice with KA-induced epilepsy as determined by quantitative analysis of immunofluorescence (Fig. 1C, D, F, G). Western blotting also showed that CXCR5 expression was increased in human epileptic tissue (Fig. 1H, I), while CXCL13 expression was not increased (Fig. 1J). Owing to sampling and ethical constraints, we used both human (cortical tissue) and C57BL/6J mouse (hippocampus) specimens to determine the pattern and localization of CXCR5 expression. Regarding subcellular localization, CXCR5 was found to mainly co-localize with Na^+^/K^+^-ATPase (Fig. 1E), indicating that CXCR5 was mainly present in neuronal membranes. Finally, cortical issue was extracted from C57BL/6J mice at different developmental stages (E14.5, E17.5, E19, P7, P14, P21, and P60) and submitted to western blot analysis. Interestingly, we found that the expression of CXCR5 was markedly reduced after birth (Fig. 1K, L), whereas that of CXCL13 remained unchanged (Fig. 1M). These findings suggested that CXCR5 might be important for embryonic development and may play a role in epilepsy.

### The Effect of the Up- or Down-regulation of *CXCR5* on Seizures

**Fig. 2:**
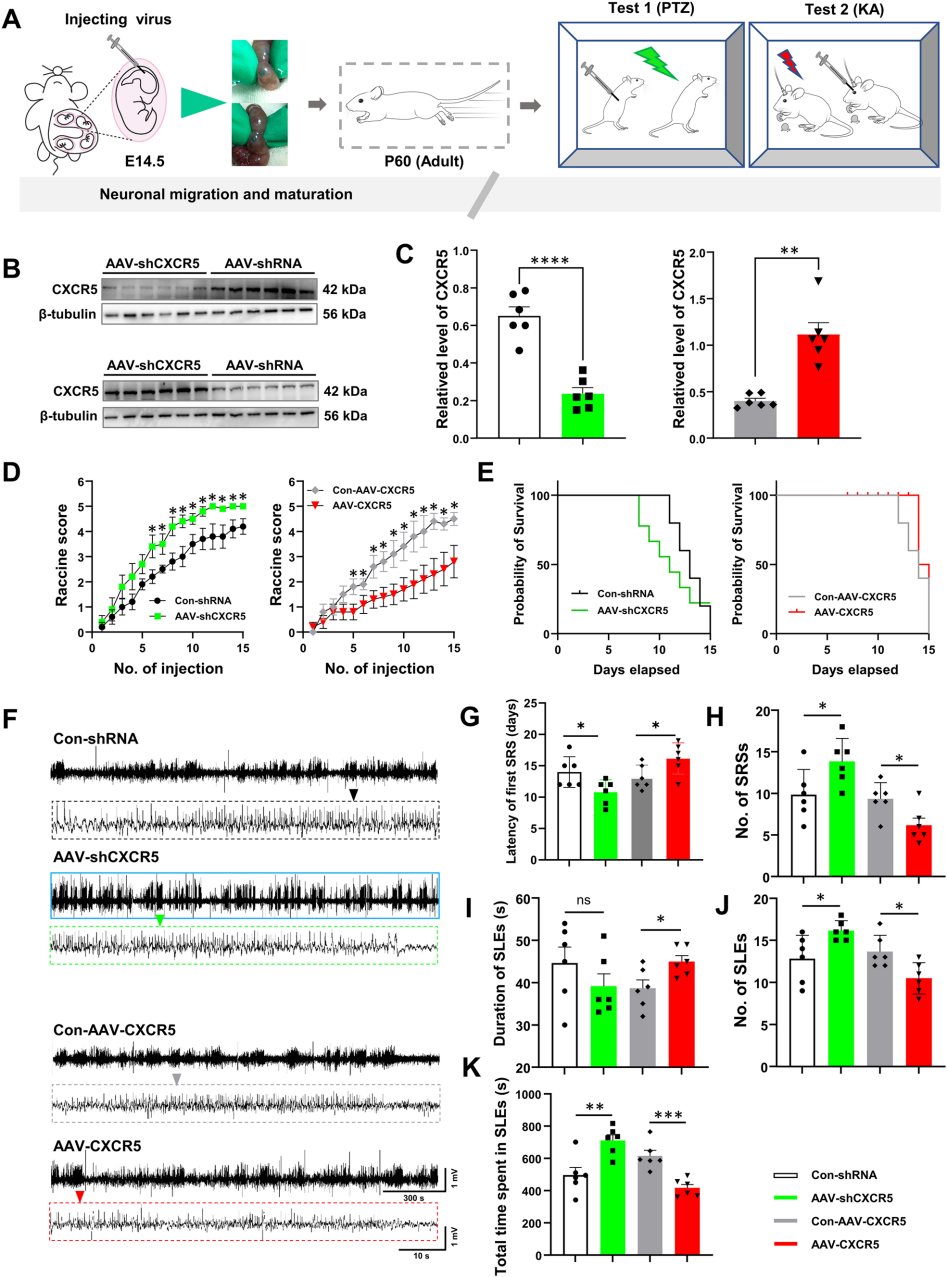
The Effect of the Up- or Down-regulation of *CXCR5* on Seizures. **A** Schematic of the experimental procedure (viral intervention of *CXCR5* at 14.5 days of the embryonic stage and behavioral testing in adult mice. **B** Western blot diagram. **C** Statistics of the effect of CXCR5 after viral intervention (*n* = 6). **D** Seizure scores of PTZ model mice (*n* = 10). **E** Survival curves of PTZ model mice (*n* = 10). **F** Representative LFPs in KA model mice. Scale as shown. **G** Latency of episodes of SRS (*n* = 6). **H** Number of episodes of SRS (1 week) (*n* = 6).** I** Duration of SLE seizures (*n* = 6).** J** Number of episodes of SLE (*n* = 6). **K** Cumulative duration of SLE episodes (*n* = 6). The data are presented as the mean ± SEM. Student’s *t*-test was used for **C** and **G**–**K**. ANOVA was used for **D** and the log-rank test was used for **E**. *P <0.05; **P <0.01; ***P <0.005; ****P <0.0001; ns, no significant difference.

Studies have associated *CXCR5* with epileptic disorders [13]. Here, we knocked down or overexpressed *CXCR5 via* the *in-utero* injection of viral vectors into the lateral ventricles of E14.5 embryos. The dark green crescent shape characteristic of the lateral ventricle observed after injection indicated that the injection was successful and could be reproduced in subsequent experiments (Fig. 2A). Injection efficiency was confirmed by western blotting (Fig. 2B, C). No spontaneous seizures were observed from birth to adulthood; accordingly, we modeled epilepsy in healthy male adult mice. PTZ-induced seizures are a relevant model of acute epilepsy. The susceptibility to subsequent epileptic seizures was explored *via* the repeated intraperitoneal injection of subthreshold doses of PTZ. The epileptic seizures of the model mice were assessed by the Racine score. On day 6, mice in the AAV-*shCXCR5* group showed higher seizure scores than those of the control group (Fig. 2D). On day 8, the *CXCR5* knockdown mice died when injected with the same dose of PTZ (Fig. 2E). On day 5, mice overexpressing *CXCR5* showed a decrease in Racine score after PTZ injection and survived until day 12 (Fig. 2D, E). This finding suggested that altering *CXCR5* gene expression can affect the susceptibility of mice to seizures; knocking down *CXCR5* increased seizure severity (higher Racine score) and mortality, whereas *CXCR5* overexpression had the opposite effect.

KA-induced seizures are among the most commonly-used models for studying the pathophysiology of epilepsy [23]. Here, healthy male mice from among breeding adults were intraperitoneally injected with KA to induce chronic SRSs. Because the epileptic effects of KA last for ~30 days [23], we conducted 1–4 weeks of continuous video surveillance and statistical analysis of recorded LFPs (Fig. 2F). We found that, compared with the control group, the latency to form SRSs decreased (Fig. 2G), whereas the number and duration of SE seizures increased (Fig. 2I) in the knockdown group; however, the opposite was seen in the AAV-*CXCR5* group (Fig. 2G, I). Finally, the cumulative seizure time for the final 7 days of video surveillance was selected for statistical analysis. The results showed that knocking down *CXCR5* increased the total SE seizure duration (Fig. 2K), whereas overexpressing *CXCR5* had the opposite effect (Fig. 2G–K). Overall, the results of both chemically-induced models of epilepsy indicated that *CXCR5* can regulate seizures.

### *CXCR5* Regulates the Motility and the Multipolar-to-Bipolar Transition of Migratory Neurons in C57BL/6J Mouse Embryos

**Fig. 3:**
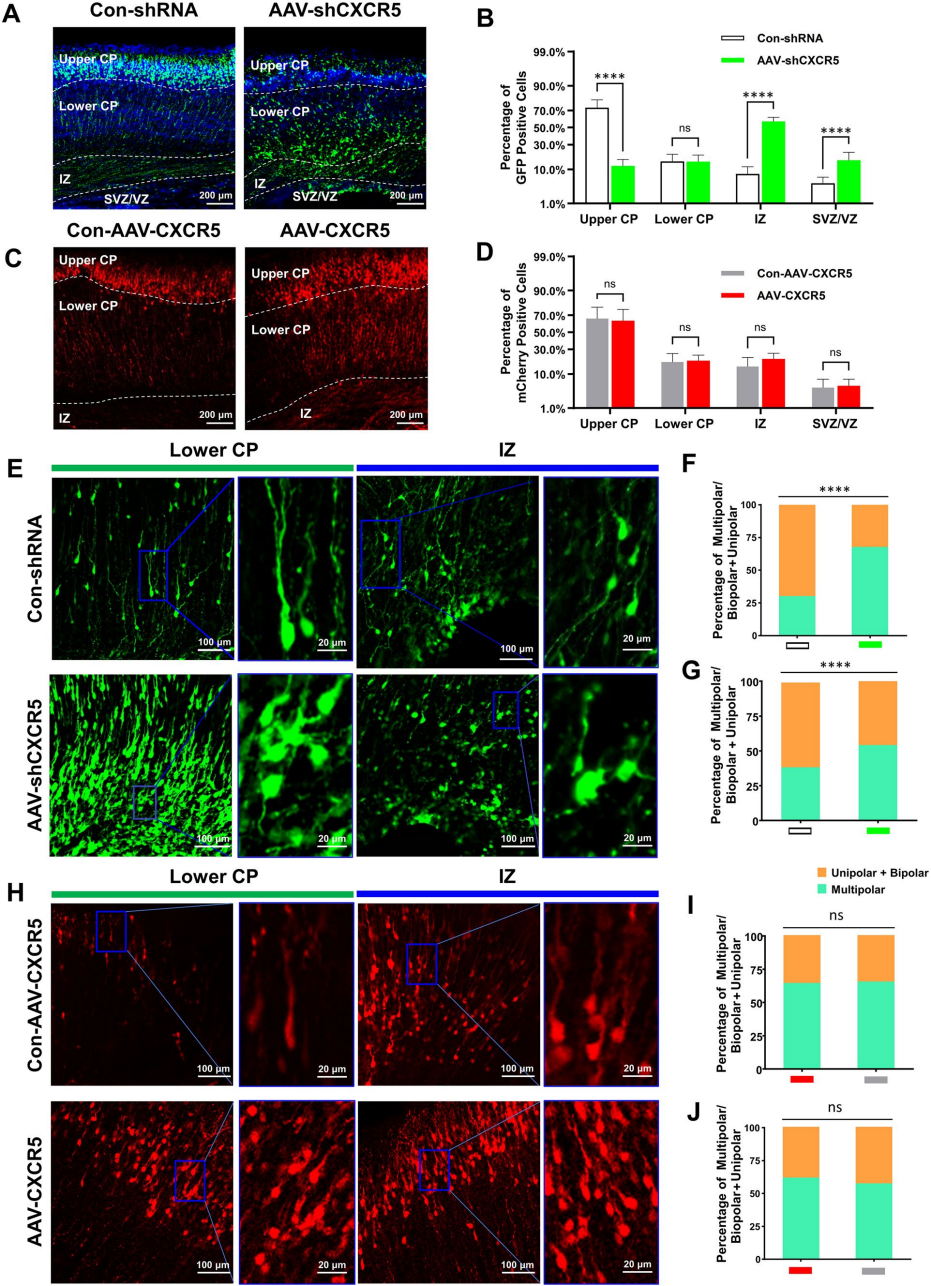
Effect of *CXCR5* on neuronal migration in murine embryos. **A** Effect of *CXCR5* knockdown on neuronal migration. Scale bars, 200 μm. **B** Proportion of green fluorescent protein (GFP) cells in different brain regions. **C** Effect of *CXCR5* overexpression on neuronal migration. Scale bars, 200 μm. **D** Proportion of red fluorescent protein (mCherry) cells in different brain regions. **E** Effect of CXCR5 knockdown on neuronal polarity in ventricular and subventricular regions. Scale bars, 100 μm and 20 μm. **F**,** G** Proportion of multipolar cells/unipolar cells in ventricular/subventricular regions in *CXCR5* knockdown and control groups; **F**, Ventricular regions; **G**, Subventricular regions. **H** Effect of *CXCR5* overexpression on neuronal polarity in ventricular and subventricular regions. Scale bars, 100 μm and 20 μm. **I**, **J** Proportion of multipolar cells/unipolar cells in ventricular/subventricular regions in *CXCR5* overexpression and control groups; **I**, ventricular regions; **J** subventricular regions. The data are presented as the mean ± SEM. *n* >10 slices from 3 mice per group. Student’s *t*-test was used for **B** and **D**. ANOVA was used for **F**, **G**,** I**, and** J**. ****P <0.0001; ns, no significant difference.

Next, we used intrauterine electroporation [24] to study the effect of *CXCR5* on neuronal migration and polarity in the embryonic period. Intrauterine electroporation was performed at E14.5 in pregnant mice to up- or down-regulate the expression of *CXCR5*, and cortical slices obtained at day 19 of gestation were stained for GFP or mCherry to assess the effects on neuronal migration (Fig. 3A, C). The distribution of GFP- or mCherry-positive cells was determined by counting the number of cells in each of the four cortical zones (from top to bottom: upper cortical plate [CP], lower CP, intermediate zone [IZ], and subventricular zone/ventricular zone [SVZ/VZ]) (Fig. 3B, D). We found that a larger proportion of cells stayed in the IZ and SVZ/VZ after AAV-*shCXCR5* compared with control groups (Fig. 3B). Next, we assessed cell polarity in two cortical regions (lower CP and IZ) and it showed distinct distributions (Fig. 3E, H). Statistical analysis of the proportions of multipolar neurons and unipolar neurons (Fig. 3F, G, I, J) revealed that the knockdown of *CXCR5* caused most of the cells to remain in the lower CP and IZ. Careful observation revealed that the direction of cell movement was inconsistent and scattered (Fig. 3E). In addition, compared with the control condition, the proportion of multipolar cells significantly increased following *CXCR5* knockdown (Fig. 3F, G). Overall, these results indicated that *CXCR5* knockdown *via* intrauterine electroporation severely affected neuronal migration and cell polarity, whereas there was no effect when *CXCR5* was overexpressed (Fig. 3D, I, J).

### The *In Vitro* Human-derived iPSC Neurodevelopmental Model and Localization of CXCR5

**Fig. 4:**
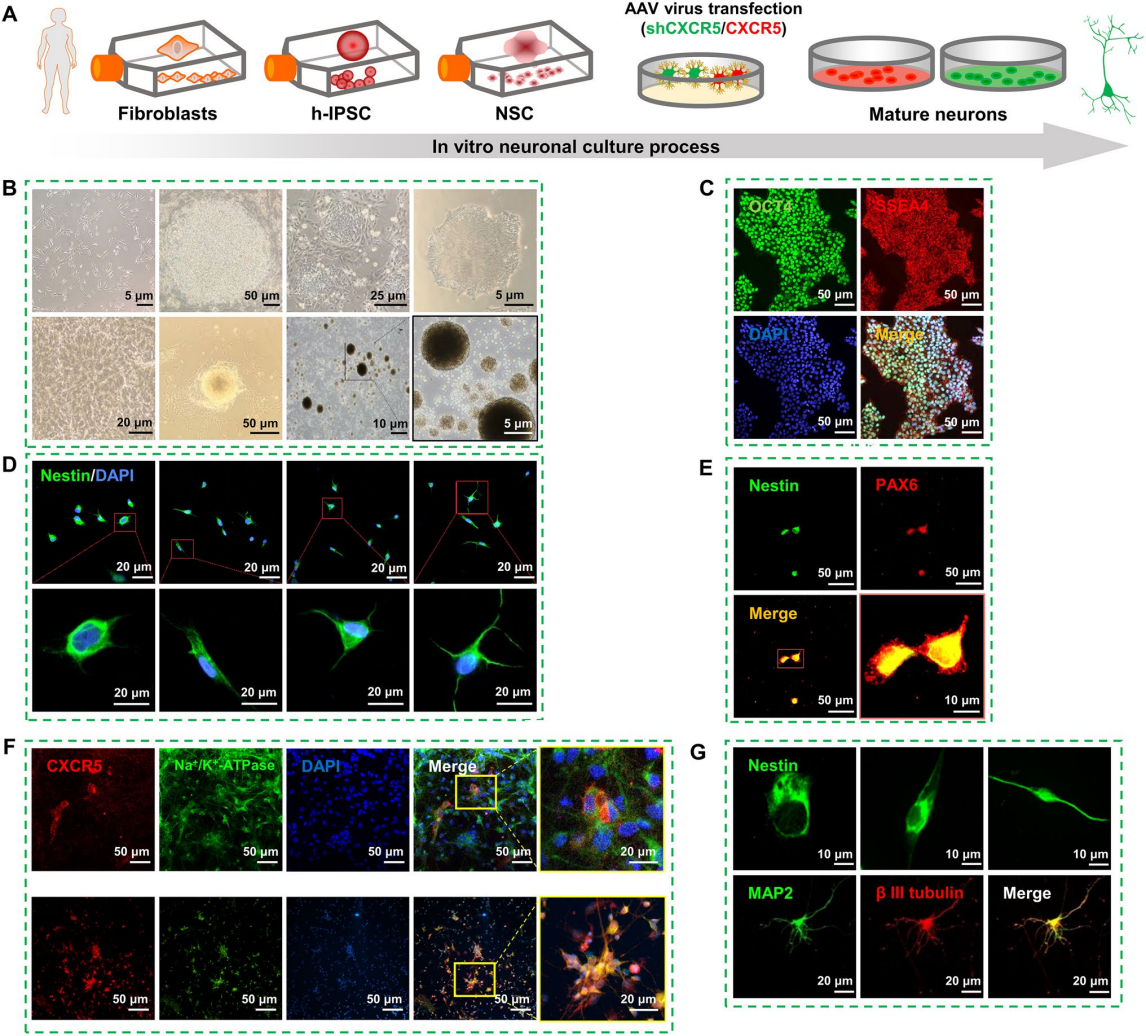
The *in vitro* human-derived iPSC neurodevelopmental model and localization of CXCR5. **A** Schematic of the induction and culture process of neurons *in vitro*. **B** Formation process of pluripotent stem cells (IPSC) and neural stem cells (NSC); primary fibroblasts (scale bar, 5 μm), successful IPSC induction (scale bar, 50 μm), IPSC passaging (scale bar, 25 μm), IPSC stabilization (scale bar, 5 μm), NSC initiation (rosette structure) (scale bar, 20 μm), NSC sphere formation (NSC prone to spherical growth) (scale bar, 50 μm), NSC suspension culture (scale bar, 10 μm), NSC spheres (scale bar, 5 μm). **C** Immunofluorescence identification of IPSCs (green, OCT4; red, SSEA4; blue, DAPI). Scale bars, 50 μm. **D** Developmental process of neurons *in vitro* (note NSC as day 0, days 1, 3, 5, and 7 are displayed from left to right). Scale bars, 20 μm. **E** Identification of NSCs (green, Nestin; red, PAX6). Scale bars, 50 μm. **F** Co-localization of CXCR5 and Na^+^/K^+^-ATPase (above are astrocytes, below are neurons) at 1 week. Scale bars, 50 μm and 20 μm. **G** Tracing morphological changes of single neurons (green, Nestin and MAP2; red, β-tubulin). Scale bars, 10 μm and 20 μm.

We applied long-term *in vitro* neuronal culture (Fig. 4A) and tracked morphological changes of individual cells under different experimental conditions. Using a human iPSC neurodevelopmental model and NSC development, embryonic somatic cells (fibroblasts) were reprogrammed to iPSCs and subsequently differentiated into mature neurons (Fig. 4B). We identified markers at different development stages, including those of iPSCs (Fig. 4C), ectodermal differentiation (Fig. 4E), neuron formation (Fig. 4D), and neuronal maturation (Fig. 4G). We applied viral intervention (*CXCR5* knockdown and overexpression) at the NSC stage and cultured the cells to neuronal maturation. To analyze the sub-localization of CXCR5 in neurons and glia, we selected neurons induced for 4 and 8 weeks. The results showed that CXCR5 co-localized well with Na^+^/K^+^-ATPase in neurons, but not in glial cells (Fig. 4F).

### The Effect of *CXCR5* on the Establishment of Polarity in Human-derived Neurons Cultured for 4 and 8 Weeks

**Fig. 5:**
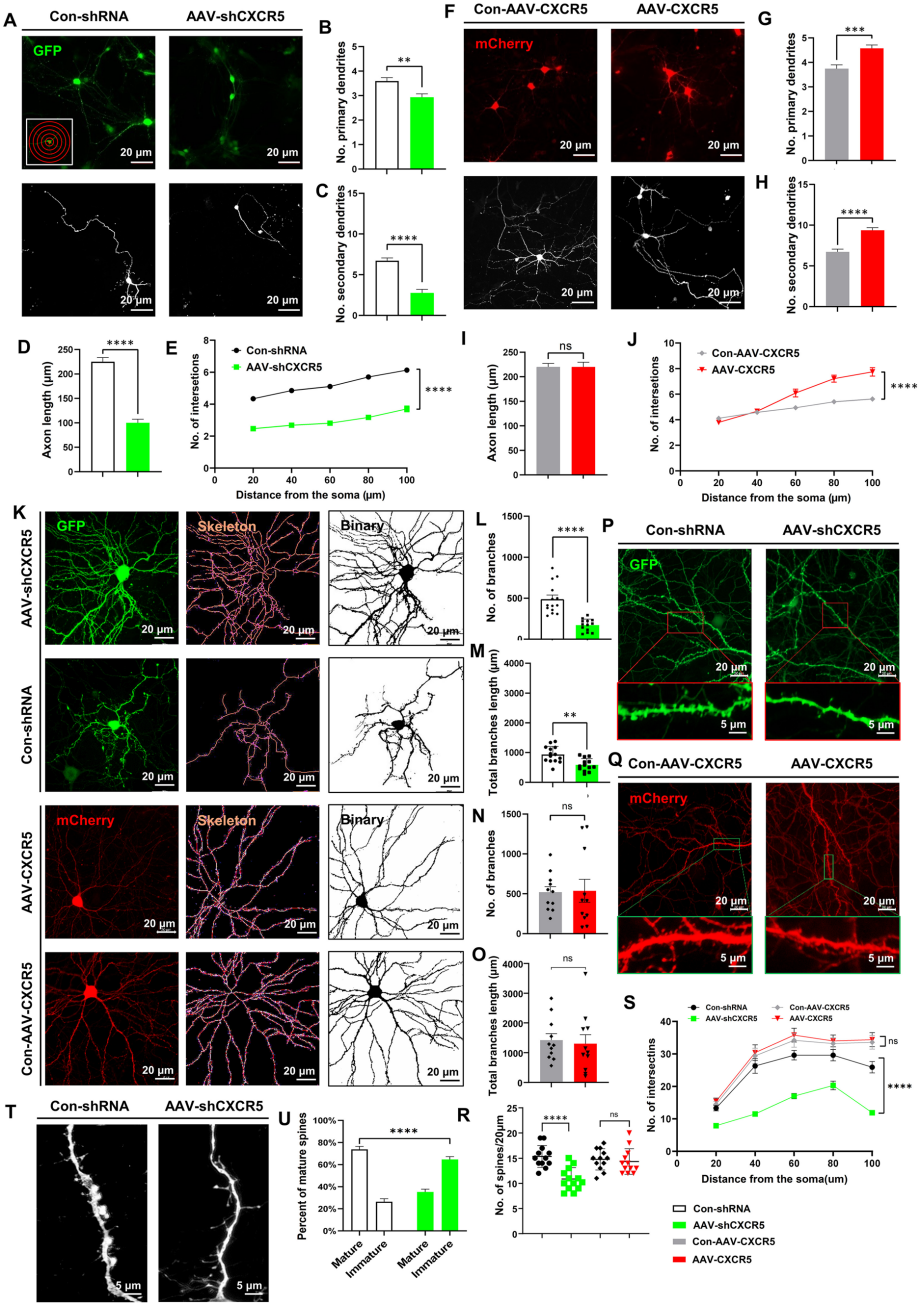
The effect of *CXCR5* on the establishment of polarity in human-derived neurons cultured for 4 and 8 weeks. **A** Representative immunofluorescence of DIV4 weeks neurons (control and knockdown *CXCR5*); left inset is a schematic of Sholl analysis. Scale bars, 20 μm. **F** Control and overexpression of *CXCR5*. Scale bars, 20 μm. **B**, **G** Statistics of numbers of primary dendrites. **C**, **H** Statistics of numbers of secondary dendrites. **D**, **I**, Statistics of axon length.** E**, **J** Statistics of the number of intersections. **K** Representative immunofluorescence, skeleton and binary images of neurons after 8 weeks in culture; scale bars, 20 μm.** L**, **N** Cell branch Statistics. **M**, **O** Total branch length statistics. **P**, **Q** Representative immunofluorescence images of dendritic spines. Scale bars, 20 μm and 5 μm. **R** Statistics of numbers of dendritic spines per 20 μm. **S** Sholl analysis statistics of DIV8 weeks. **T** Representative immunofluorescence images of dendritic spine maturity [*left*, mature dendritic spines (mushroom-like); *right*, immature dendritic spines (filamentous pseudopods)]. Scale bars, 5 μm.** U** Statistics of the proportion of mature dendritic spines. The data are presented as the mean ± SEM. *n* = 47. Student’s *t*-test was used for **B**, **C**, **D**, **G**, **H**, **I**, **L**, **M**, **N**, **O**, **R**, and** U**; ANOVA was used for **E**, **J**, and** S**. **P <0.01; ***P <0.005; ****P <0.0001; ns, no significant difference.

At 4 weeks in culture, compared with the control group (Fig. 5A), the number of primary and secondary neurites was reduced in the AAV-*shCXCR5* group (Fig. 5B, C) but increased in the AAV-*CXCR5* group (Fig. 5F, G, H). Sholl analysis [25] indicated that the knockdown of *CXCR5* resulted in a significant reduction in neuronal complexity (Fig. 5E), while in the AAV-*CXCR5* group it was increased (Fig. 5J). We further found that knocking down *CXCR5* led to a reduction in neuronal axon length (Fig. 5D), whereas axon length was not affected in the group overexpressing *CXCR5* (Fig. 5I). At week 8, we analyzed the neuronal skeleton (Fig. 5K), and found that, compared with the control group, knocking down *CXCR5* significantly reduced the total number of neuronal branches as well as total branch length (Fig. 5L, M); however, overexpressing *CXCR5* did not affect these parameters (Fig. 5N, O). We also applied Sholl analysis at week 8, and showed that the knockdown of *CXCR5* significantly reduced neuronal complexity (Fig. 5S). Subsequent statistical analysis showed that dendritic spine density was significantly lower in the knockdown group than in the control group (Fig. 5P, R); However, overexpression of *CXCR5* had no effect. (Fig. 5Q, R). Moreover, we found that the percentage of immature dendritic spines increased significantly after the knockdown of *CXCR5* (Fig. 5T, U).

Analysis of neuronal morphology at these two developmental stages revealed that the knockdown of *CXCR5* profoundly affected neuronal development, including hindering the formation of dendrites and axons and the maturation of the dendritic spine. Interestingly, *CXCR5* overexpression promoted early neuronal growth at week 4 but did not affect neuronal maturation at week 8.

### *CXCR5* Regulates Neuronal Migration and Polarization by Stabilizing the Actin Cytoskeleton

**Fig. 6:**
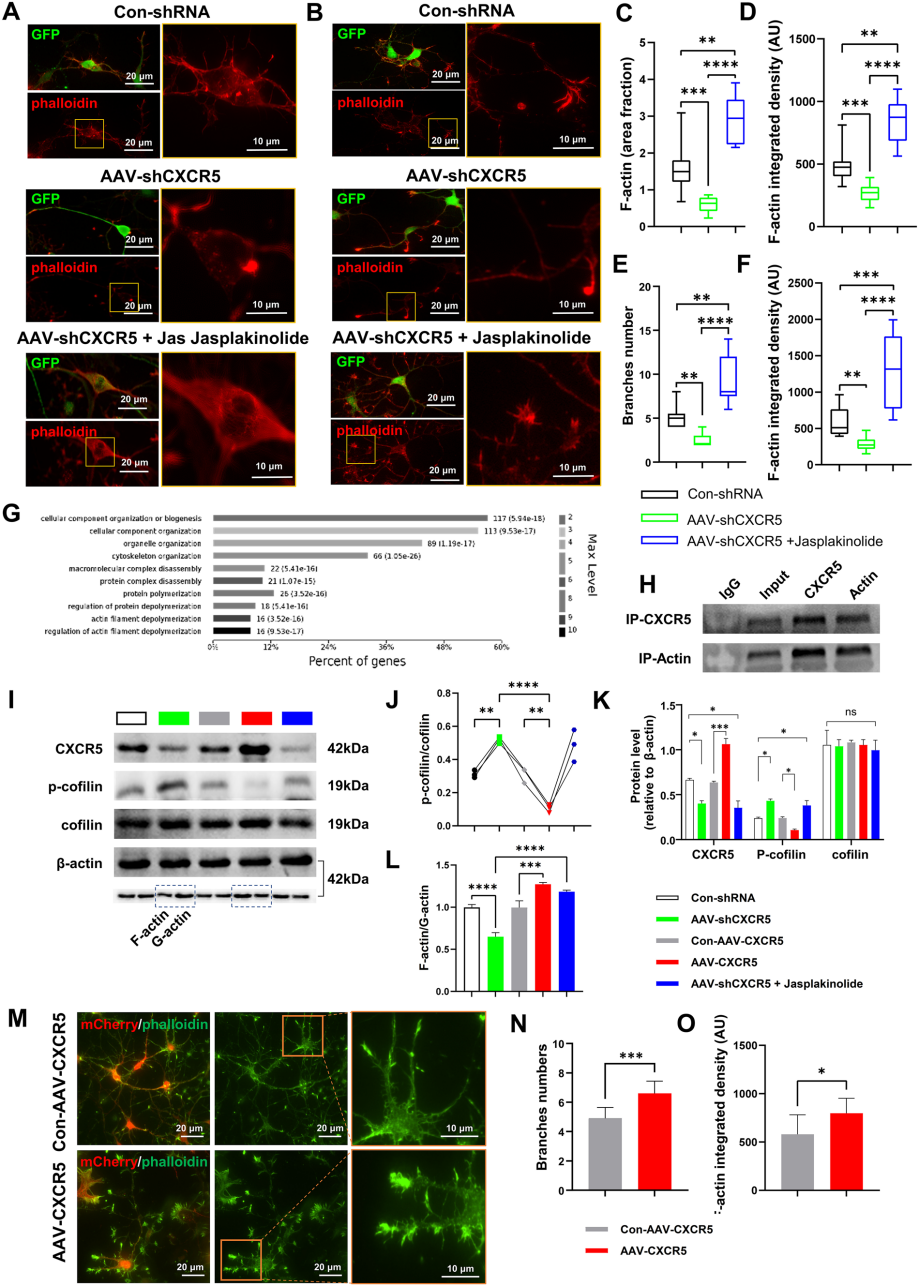
*CXCR5* Regulates Neuronal Migration and Polarization by Stabilizing the Actin Cytoskeleton. **A**, **B** Representative immunofluorescence images of CXCR5 neuron (green) and phalloidin (red) in three groups (Con-shRNA, AAV-*shCXCR5,* and AAV-*shCXCR5* + Jas). Scale bars, 20 μm and 10 μm. **C**, **D** Fluorescence area ratio statistics and total fluorescence analysis of F-actin in the three groups corresponding to **A** (*n* = 10). **E** Statistics of branch numbers in the three groups corresponding to **B** (*n* = 10). **F** Statistics of F-actin fluorescence in the three groups corresponding to **B** (*n* = 10). **G** CXCR5 enrichment analysis (BF) results in the protein profile. **H** Images of immunoprecipitation of CXCR5 and actin. **I** Representative western blot images of CXCR5, p-cofilin, and cofilin in five groups (Con-shRNA, AAV-*shCXCR5*, Con-AAV-*CXCR5*, AAV-*CXCR5*, and AAV-*shCXCR5* + Jas). **J** Protein levels of p-cofilin/cofilin in the five groups corresponding to **I**. **K** Protein levels of CXCR5/β-actin, p-cofilin/β-actin, and cofilin/β-actin in the five groups corresponding to **I**. **L**, Protein levels of F-actin/G-actin in the five groups corresponding to **I**. **M** Representative immunofluorescence images of mCherry (red) and phalloidin (green) in two groups (Con-AAV-*CXCR5* and AAV-*CXCR5*). Scale bars, 20 μm and 10 μm. **N** Statistics of branch numbers in the two groups corresponding to **M** (*n* = 10). **O** Statistics of F-actin fluorescence in the two groups corresponding to **M** (*n* = 10). The data are presented as the mean ± SEM. The Kruskal–Wallis test was used for **C**−**F**; ANOVA was used for **J**−**L**. The independent Student’s *t*-test was used for** N** and **O**; *P <0.05; **P <0.01; ***P <0.005; ****P <0.0001; ns, no significant difference. Jas, Jasplakinolide.

We found that CXCR5 protein expression peaked at embryonic stage E17.5 and gradually decreased after birth. Accordingly, we extracted mouse brain cortical proteins at E17.5 and undertook a protein interaction assay (co-immunoprecipitation; co-IP) for CXCR5, followed by protein profiling. We applied bioinformatic analysis to these reciprocal proteins (GO function and KEGG pathway enrichment analysis), and found that most of the reciprocal proteins were associated with the intracellular skeleton (Fig. 6G). Then examining the protein interaction between CXCR5 and actin by co-IP experiments (Fig. 6H).

Focusing on actin and cofilin, we next investigated the mechanism by which *CXCR5* regulates neuronal migration and neurite growth. The actin cytoskeleton was stained with rhodamine-labeled phalloidin *in vitro* for 4 weeks. Growth cones were evident in neurons in the AAV-*CXCR5* group, and F-actin accumulation was observed at the leading edge (Fig. 6A, B), F-actin/G-actin distribution and expression were determined by quantitative fluorescence microscopy and Western blot analysis. The results showed that knocking down *CXCR5* reduced the fluorescence range and fluorescence intensity of F-actin (Fig. 6C, D) (the exposure intensity was not changed during fluorescence imaging, and the threshold segmentation was uniformly set between 20 and 255 for statistical analysis), while F-actin coverage increased with overexpression of *CXCR5* (Fig. 6M−O). Interestingly, the fluorescence range and fluorescence intensity of F-actin were re-elevated by jasplakinolide (Fig. 6C–F), an F-actin agonist. Furthermore, we found that *CXCR5* knockdown increased the phosphorylation of the F-actin-removing factor cofilin (Fig. 6I–L). As expected, overexpression of *CXCR5* resulted in an increased F-actin/G-actin ratio due to decreased phosphorylation of cofilin (Fig. 6I–L); interestingly, the same effect occurred using jasplakinolide (Fig. 6I–L).

### The Effect of *CXCR5* on Neuronal Excitability and Drug Rescue Experiments in the Embryonic Stage and Neuronal Maturation *In Vitro*

**Fig. 7:**
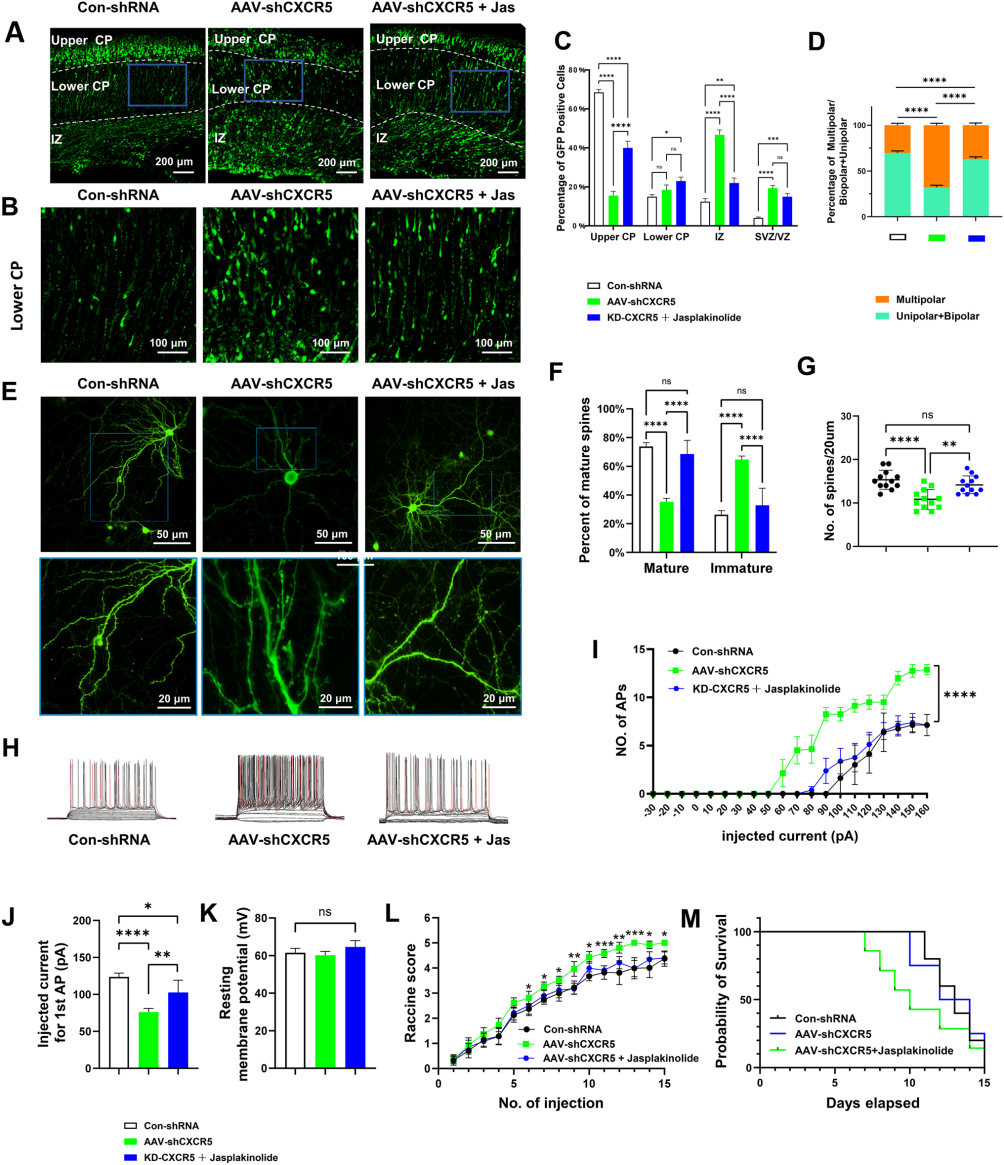
The effect of *CXCR5* on neuronal excitability and drug rescue experiments in the embryonic stage and *in vitro* neuronal maturation. **A** Representative immunofluorescence images of Con-*shRNA*, AAV-*shCXCR5,* and AAV-*shCXCR5* + Jas during neuronal migration in the upper CP, lower CP, and IZ regions. Scale bars, 200 μm. **B** Representative immunofluorescence images of Con-shRNA, AAV-*shCXCR5,* and AAV-*shCXCR5* + Jas during neuronal migration in the lower CP region; scale bars, 100 μm. **C** Proportion of green fluorescent protein (GFP) cells in different brain regions (*n* = 10). **D** Proportion of multipolar cells/unipolar cells in the lower CP region (*n* = 10). **E** Representative immunofluorescence images of con-shRNA, AAV-*shCXCR5,* and AAV-*shCXCR5* + Jas in the growth of mature neurons. **F** Statistics of the proportions of mature dendritic spines (*n* = 10). G Numbers of dendritic spines per 20 μm (*n* = 10). **H** Representative images of evoked action potentials in three groups (Con-shRNA, AAV-*shCXCR5,* and AAV-*shCXCR5* + Jas). **I** The firing frequency distribution of evoked action potentials of neurons under different voltage stimulation in the three groups corresponding to **H** (*n* = 10). **J** Stimulated current for the first action potential of neurons in the three groups corresponding to **H** (*n* = 10). **K** Resting membrane potential of neurons in the three groups corresponding to **H** (*n* = 10). **L** Seizure scores of PTZ model mice with con-shRNA, AAV-*shCXCR5,* and AAV-*shCXCR5* + Jas (*n* = 12). **M** Survival curves of PTZ model mice corresponding to **L** (*n* = 12). ANOVA was used for **C**, **F**,** I**, and** L**; the Kruskal–Wallis test was used for **D**,** G**,** J**, and** K**; The log-rank test was used for **M**. *P <0.05; **P <0.01; ***P <0.005; ****P <0.0001; ns, no significant difference. Jas, Jasplakinolide.

The above results indicated that (1) *CXCR5* deficiency leads to impaired migration of late-born embryonic neurons (*in utero* electroporation experiments) and morphological defects during development (*in vitro* iPSC neurodevelopmental model); (2) the up- or down-regulation of *CXCR5* leads to increased seizure activity in adult mice (as exemplified by two models of epilepsy [KA- and PTZ-induced]). To explore why the knockdown of *CXCR5* in cortical neurons leads to higher susceptibility to epilepsy, we tested neuronal excitability using whole-cell patch-clamp recording in neurons *in vitro*, and found that the resting membrane potential was unaffected (Fig. 7H, K). However, knocking down *CXCR5* increased the evoked spike frequency (Fig. 7L) and changed the pattern of eAPs (Fig. 7J, L). This indicated that *CXCR5* deficiency led to firing instability in these neurons. Combined, these findings suggested that the higher susceptibility to epilepsy in *CXCR5*-deficient mice may be due to increased neuronal firing that might be associated with abnormal firing patterns.

To elucidate the causal relationship between *CXCR5* and epilepsy, we performed drug rescue experiments both *in vivo* and *in vitro*. We found that the addition of jasplakinolide alleviated the impairment of neuronal migration when *CXCR5* was knocked down, resulting in increased upward migration of neurons from the lower CP region, with more neurons detected in the upper CP (Fig. 7A–D). In addition, jasplakinolide reversed the side-effect that the percentage of multipolar cells in the lower CP region increased with *CXCR5* deficiency (Fig. 7A–D). We also found that jasplakinolide counteracted the side-effects of *CXCR5* deficiency in neuronal development (Fig. 7E–G) and the firing instability of *CXCR5* deficiency *in vitro* (Fig. 7H–K). resulting in reduced seizure scores in PTZ-induced animals with *CXCR5* deficiency (Fig. 7L, M).

## Discussion

Epilepsy is a severe neurological disorder characterized by recurrent spontaneous seizures that affect 1% of the global population [1]. In this study, we discovered novel functions of *CXCR5* and its role in epilepsy and attempted to determine its underlying mechanism.

Owing to sampling and ethical constraints, we used both human and mouse specimens to determine the pattern and localization of CXCR5 expression. We found that CXCR5 was predominantly expressed in the embryonic stage in mice, which is a novel finding. We further found that CXCR5 was mainly distributed in the cortex, and mainly localized to neuronal membranes, which is consistent with previous findings [13].

In addition to genetic modification, two epilepsy modeling approaches are frequently used in animals, namely, (1) acute epilepsy models, in which a chemical (such as PTZ), an acoustic source, or an electrical stimulus, is applied; and (2) chronic seizure models, in which KA or trichothecene are applied to induce SRSs following the structural damage caused by sustained SE [26, 27]. As no spontaneous seizures were observed in mice from birth to adulthood with either loss or gain of function of the *CXCR5* gene, we established KA- and PTZ-induced epilepsy models in adult mice and monitored their susceptibility to seizures. The results showed that embryonic *CXCR5* depletion led to higher susceptibility to seizures in adult mice, while *CXCR5* overexpression had the opposite effect.

The normal functioning of the nervous system depends on its conventional structural development. In the mouse embryo, most cortical neurons (pyramidal neurons) are born in the VZ *via* asymmetric division of radial glial cells and migrate along the radial projections of glial cells toward specific regions of the peripheral cortex during the late stage of cerebral cortex development [28]. Our experiments based on intrauterine electroporation are the first to reveal that the knockdown of *CXCR5* during the longitudinal migration of cortical neurons from the SV/SVZ region to the upper CP in late gestation results in multipolarity and disturbed direction of migration, with most of the cells staying in the SVZ. This evidence suggests that *CXCR5* deficiency causes a disorder of neuronal migration in the late embryonic period.

To scrutinize the effect of *CXCR5* on neuronal polarity, we performed *in vitro* cell culture experiments based on a neurodevelopmental model of human-derived iPSCs [29] to simulate human nervous system development. During the development of the nervous system, neurons generated in the ventricular and subventricular layers must migrate to specific brain regions to exert their function. Neuronal migration is essential for the normal development of brain circuits and functional brain activity. Here, we found that *CXCR5* deficiency affects epilepsy by contributing to impaired neuronal migration during the embryonic period. However, animal models of neurodevelopment cannot fully replicate the human condition [30, 31]. Accordingly, we established a neurodevelopmental model using human-derived iPSCs [30] that can, to a certain extent, replicate the environment of human embryonic development. We found that, following the knockdown of *CXCR5*, the axial dendrites of neurons exhibited impaired development compared with the control group at week 4 of induced differentiation. By week 8, a marked reduction in neuronal complexity was evident. Furthermore, we found that the knockdown of *CXCR5* hindered neuronal maturation, as evidenced by reduction in total branch number and total branch length, as well as decreased dendritic spine density and impaired dendritic spine maturation. In the mammalian cerebral cortex and hippocampus, dendritic spines normally receive and integrate excitatory synaptic signals, and abnormal dendrites may lead to excessive neuronal firing. In summary, abnormal dendritic spine development can directly cause neuronal excitability and seizures. To demonstrate that these effects of *CXCR5* deficiency are specific to neurons and not to other neuronal cells, we overexpressed *CXCR5* under the control of a neuron-specific promoter using a viral vector. We found that the overexpression of *CXCR5* led to a significant increase in the number of primary and secondary neuropil synapses in neurons at week 4, but did not exert a positive effect at week 8. These findings fully validate and complement the results obtained *in vivo* in mouse embryos. Our results confirmed that *CXCR5* gene deficiency causes abnormal neuronal development and polarity, impairs neuronal migration in late pregnancy, and ultimately increases the susceptibility to epilepsy in adulthood. To determine how CXCR5, a G protein-coupled receptor localized on the cell membrane, affects neuronal polarity, we focused on actin proteins based on our protein profiling results. Interruption of the dynamic of F-actin/G-actin cycle leads to an imbalance in actin aggregation and depolymerization that can lead to amyloid degeneration, which is found in Down syndrome and Alzheimer’s disease. These consequences stem from the fact that actin is essential for cortical development and dendritic spine formation [30]. This pathogenicity may occur during development and manifest as a disease in the adult. In the AAV-*shCXCR5* group, F-actin had a lower expression level and range, and the neurons showed a more naive state than that seen in the control group. We quantified F-actin by immunofluorescence and western blot with strict homogeneous control of exposure intensity and segmentation threshold; using jasplakinolide recovered abundant myofilament proteins and dendritic branches in *CXCR5*-deficient neurons. Our results reinforce the idea that the disruption of microtubule-based processes underlies widespread neuronal migration disorders, and further suggest that *CXCR5* regulates neuronal migration and polarization by stabilizing the actin cytoskeleton.

Our findings suggested that the knockdown of *CXCR5* during embryonic development is associated with seizures in adulthood. Susceptibility to epilepsy has long been thought to be related to the stability of neural networks and intrinsic neuronal electrophysiological homeostasis. Our data suggest that *CXCR5* may promote the survival of abnormally excitable neurons resulting from developmental migration disorders, leading to increased epilepsy susceptibility.

In conclusion, our *in vivo* and *in vitro* experiments, which included behavioral experiments, *in utero* electroporation, cell culture, and electrophysiological experiments, demonstrated that *CXCR5* deficiency during embryonic development leads to abnormal neuronal polarity, impaired neuronal migration, and increased neuronal hyperexcitability, resulting ultimately in increased susceptibility to epilepsy and seizures.

Almost all neuronal migration disorders, including anencephaly, periventricular nodal ectopia, and subcortical zone ectopia, are risk factors for seizures [32]; however, the exact mechanisms *via* which they cause epileptiform activity are unknown. Focal cortical dysplasia is estimated to be associated with 30% of cases of refractory epilepsy, but neither surgical nor pharmacologic treatment is satisfactory in these patients [33]. Furthermore, antiseizure medications, which function by inhibiting neuronal excitation or enhancing neuronal inhibition, act indiscriminately throughout the nervous system and thus exert a wide range of adverse effects. Our findings provide a different perspective on *CXCR5* gene-targeted therapy for refractory epilepsy.

### Supplementary Information

Below is the link to the electronic supplementary material.

Supplementary file1 (PDF 145 kb)
