# Supplementary material for: CXCR5 Regulates Neuronal Polarity Development and Migration in the Embryonic Stage via F-Actin Homeostasis and Results in Epilepsy-Related Behavior
Source: Neurosci Bull. 2023 Jul 17;39(11):1605–22. doi: 10.1007/s12264-023-01087-w (PMC10603003; doi:10.1007/s12264-023-01087-w)
Supplement: Supplementary file 1 — Supplementary file1 (PDF 145 kb) [file 12264_2023_1087_MOESM1_ESM.pdf]

Supplementary Material

Table S1 Specific information of plasmid vectors, viral vectors, antibodies, and reagents

Reagent		Source	Identifier
**Adeno-associated Virus**			
pAAV-U6-shRNA(CXCR5)-CMV-EGFP-WPRE			
pAAV-U6-shRNA(NC2)-CMV-EGFP-WPRE		OBiO	
pAAV-hysn-CXCR5-mCherry-WPRE		Technology	
pAAV-hysn-mCherry -WPRE			
**Plasmid**			
pAAV-U6-shRNA(Cxcr5)-CMV- EGFP-WPRE			
pAAV-U6-shRNA(NC2)-CMV-EGFP-WPRE		OBiO	
pAAV-hSyn-mCherry-P2A-Cxcr5-3xFLAG- WPRE		Technology	
pAAV-hSyn-mCherry-P2A-3xFLAG-WPRE			
**Antibody**			
CXCR5 Rabbit mAb	1:1000	ZENBIO	Cat# R24019
CXCR5 mouse mAb	1:1000	Santa Cruz	Cat# sc-373775
Beta tubulin mouse mAb	1:4000	Proteintech	Cat#66009-1-lg
Beta-actin rabbit pAb	1:5000	Proteintech	Cat# 20536-1-AP
Phospho-Cofilin (Ser3) Rabbit pAb	1:500	ZENBIO	Cat# 310036
Cofilin Rabbit pAb	1:500	ZENBIO	Cat# 310140
Anti-F-actin mouse mAb	1:1000	Abcam	Cat# ab205
CXCL13 rabbit pAb	1:1000	GeneTex	Cat# GTX108471
Anti-CXCR5 rabbit mAb	1:5000	Abcam	Cat# ab254415
GFAP mouse mAb	1:2000	ZENBIO	Cat# 250027
MAP2 mouse mAb	1:2000	ZENBIO	Cat# 250035
Na^+/K^+-ATPase mouse mAb	1:200	SANTA CRUZ	Cat# sc-514614
Anti-mCherry mouse pAb	1:500	Proteintech	Cat# 26765-1-AP
Anti-GFP mouse pAb	1:500	Abcam	Cat# ab1218
Oct-4 Rabbit pAb	1:500	ZENBIO	Cat# 381335
SSEA4 mouse mAb	1:500	Abcam	Cat# ab16287
Nestin rabbit pAb	1:500	ZENBIO	Cat# 381211
PAX6 mouse mAb	1:500	Proteintech	Cat# 6964-1-lg
GFP mouse mAb	1:500	Abcam	Cat# ab1218
mCherry rabbit pAb	1:500	Proteintech	Cat# 26765-1- AP
HRP-conjugated Affinipure Goat Anti-Mouse IgG (H+L)	1:5000	Proteintech	Cat# SA00001-1
HRP-conjugated Affinipure Goat Anti-Rabbit IgG (H+L)	1:400	Proteintech	Cat# SA00001-2
CoraLite594-conjugated Donkey Anti-Rabbit IgG (H+L)	1:400	Proteintech	Cat #SA00013-8
CoraLite488-conjugated Donkey Anti-Mouse IgG (H+L)		Proteintech	Cat #SA00013-5

**Cell Culture**		
DMEM/F-12	Gibco	Cat #11320033
Human serum	Sigma	Cat # SLCF0694
Penicillin/streptomycin/neomycin	Gibco	Cat # 15640055
NutriFreez D10 Cryopreservation Medium	Biological industries	Cat# 2144029
Accutase	STEMCELL	Cat #07920
EDTA	Invitrogen	Cat# 01032272
BD Matrigel hESC-qualified Matrix	Biological Industries	Cat# 354227
Laminin	Sigma-Aldrich	Cat# 114956-81-9
poly-D-lysine	Sigma-Aldrich	Cat# 27964-99-4
NutriStem hPSC XF Medium	Biological Industries	Cat# 2132733
StemRNA 3rd Gen Reprogramming Kit for Reprogramming Adult and Neonatal Human Fibroblasts	Stemgent	Cat# 00-0076
Lipofectamine RNAiMAX Transfection Reagent	Invitrogen	Cat# 2341058
LDN193189	MedChemExpress	Cat# HY-12071
SB431524	MedChemExpress	Cat# HY-12848
N2 Supplement	Gibco	Cat# 17502001
B27 supplement	Gibco	Cat# 2313316
Neurobasal Medium	Gibco	Cat# 21103049
Insulin-Transferrin-Selenium-Sodium Pyruvate (ITS-A)	Gibco	Cat# 51300044
GlutaMAX Supplement	Gibco	Cat# 35050061
Human FGF-basic (FGF-2/bFGF)	Gibco	Cat# 13256-029
EGF	Gibco	Cat# PHG0314
96-well Ultra-Low Attachment Microplate	Corning	Cat# 7007
**Animal reagents**		
Pentylenetetrazole	Sigma–Aldrich	Cat# 54-95-5
kainic acid	Sigma–Aldrich	Cat# 487-79-6
Jasplakinolide	Sigma-Aldrich	Cat# 102396-24-7
